# Supplementary material for: A Novel Role of the Two-Component System Response Regulator UvrY in Enterohemorrhagic Escherichia coli O157:H7 Pathogenicity Regulation
Source: Int J Mol Sci. 2023 Jan 24;24(3):2297. doi: 10.3390/ijms24032297 (PMC9916836; doi:10.3390/ijms24032297)
Supplement: Supplementary file 1 [file ijms-24-02297-s001.zip › Supplementary materials.pdf]

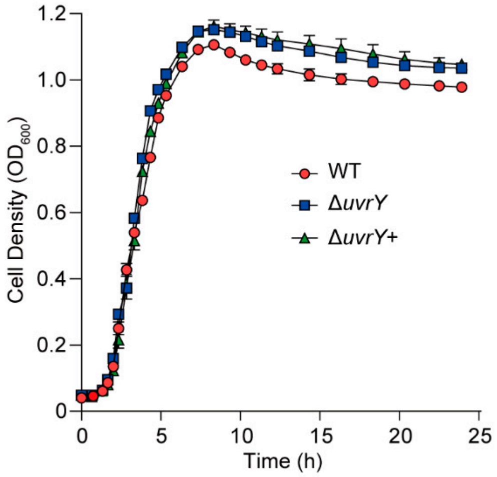


**Figure S1.** The growth curves of WT, Δ*uvrY*, and Δ*uvrY*+ strains in LB broth (*n*=3).

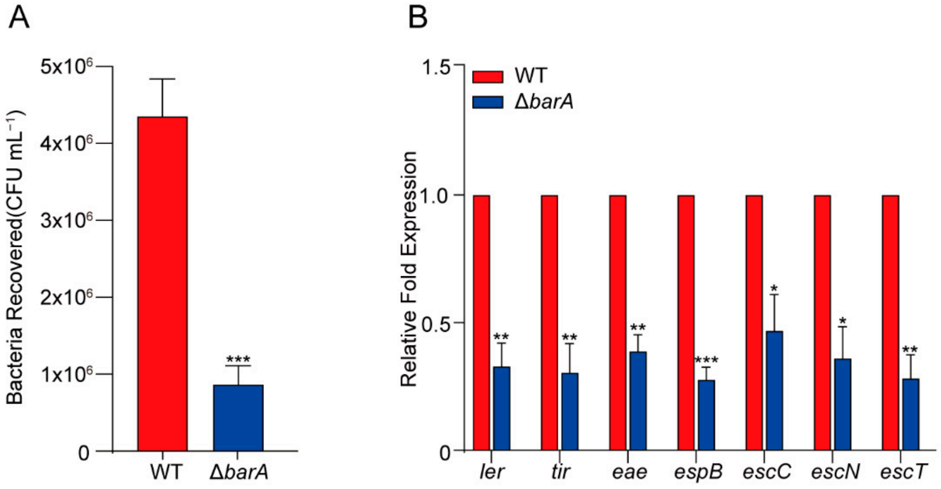


**Figure S2.** BarA positively affects the virulence of EHEC O157:H7. (A) Adhesion of WT and Δ*barA* to HeLa cells. (B) qRT-PCR of LEE genes expression changes in WT and Δ*barA* strains.

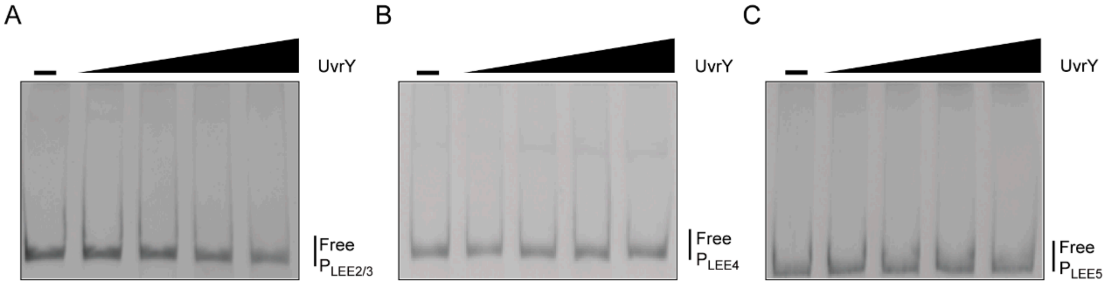

**Figure S3.** EMSA showing the in-vitro UvrY binding activity. (A–C) Chemiluminescence photographs showing the EMSA result for the binding affinity of protein UvrY-His6 with (A) the LEE2/3 promoter; $P_{LEE2/3}$, (B) LEE4 promoter; $P_{LEE4}$ and (C) LEE5 promoter; $P_{LEE5}$. 30 ng of PCR products were added to each DNA and UvrY-his6 protein reaction mixtures from lane 1-5 in A-C. No protein was added to lane 1, while lane 2-5 contains reaction mixtures with UvrY-his6 protein concentrations from low to high, accordingly.

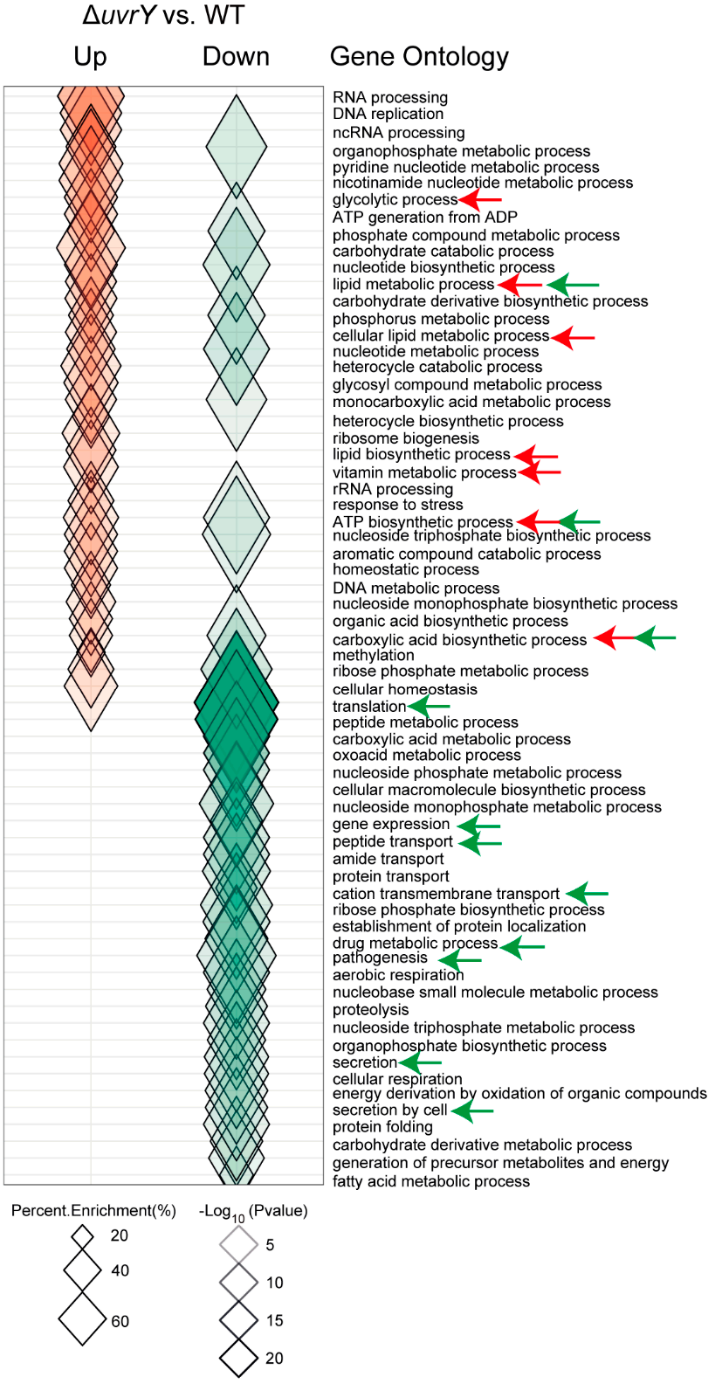


**Figure S4.** GO analysis of UvrY-regulated genes in EHEC O157:H7. Bubble plots showing the enriched GO results for down- and up-expressed genes in Δ*uvrY* strains compared with WT.

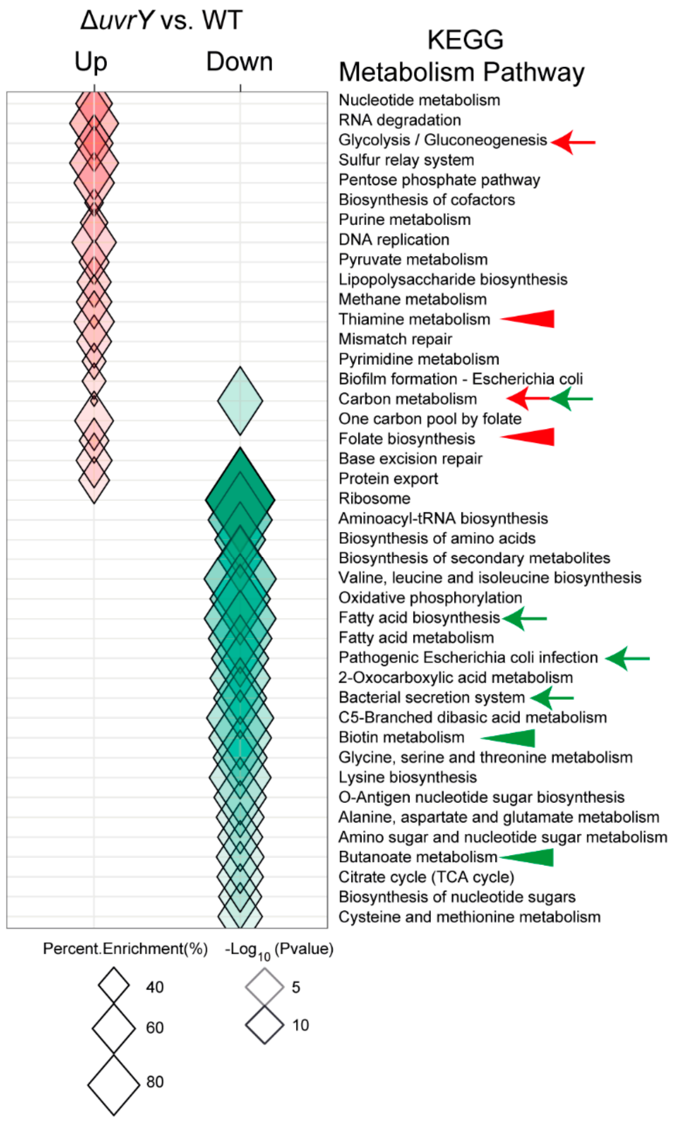


**Figure S5.** KEGG analysis of UvrY-regulated genes in EHEC O157:H7. Bubble plots showing the KEGG Pathway enrichment results for down- and up-expressed genes in Δ*uvrY* strains compared with WT.
