# Supplementary material for: A Novel Role of the Two-Component System Response Regulator UvrY in Enterohemorrhagic Escherichia coli O157:H7 Pathogenicity Regulation
Source: Int J Mol Sci. 2023 Jan 24;24(3):2297. doi: 10.3390/ijms24032297 (PMC9916836; doi:10.3390/ijms24032297)
Supplement: Supplementary file 1 [file ijms-24-02297-s001.zip › Table S2.pdf]

Table S2. Strains and plasmids used in this study

Strains	Genotype or description	Source or reference
G2734	Wild-type EHEC O157:H7 EDL933	ATCC*
Δ*uvrY*	*uvrY* deletion mutant in G2734	This study
Δ*ler*	*ler* deletion mutant in G2734	This study
Δ*barA*	*barA* deletion mutant in G2734	This study
Δ*ler*Δ*uvrY*	*ler* and *uvrY* double deletion mutant in G2734	This study
Δ*uvrY*+	Δ*uvrY* containing pTrc99a-*uvrY*	This study
Δ*ler*Δ*uvrY*+	Δ*ler*Δ*uvrY* double deletion mutant containing pTrc99a-*uvrY*	This study
uvrY-6 x His	BL21(DE3) containing pET28a-*uvrY*	This study
uvrY-3 x FLAG	Δ*uvrY* containing pTRC99a-*uvrY*-3×FLAG	This study
Plasmids		
pKD3	Containing a chloramphenicol resistance cassette and the flipase recognition sites, CmR	BfR†
pKD4	Containing a kanamycin resistance cassette and the flipase recognition sites, KmR	BfR†
pKD46	Red recombination plasmid, ApR	Lab collection
pET28a	T7 expression vector, KmR	Lab collection
pTRC99a	Expression vector, ApR	Lab collection
pTRC99a-*uvrY*-3×FLAG	pTRC99a carrying *uvrY*-3x FLAG, ApR	This study
pTRC99a-*uvrY*	pTRC99a carrying *uvrY*, ApR	This study
pET28a-*uvrY*	pET28a carrying *uvrY*, KmR	This study

*, ATCC, American Type Culture Collection, Manassas, Virginia, USA. †, BfR, Beutin Federal institute for Risk Assessment National Reference Laboratory for Escherichia coli, Berlin, Germany.
