# Supplementary material for: A Novel Role of the Two-Component System Response Regulator UvrY in Enterohemorrhagic Escherichia coli O157:H7 Pathogenicity Regulation
Source: Int J Mol Sci. 2023 Jan 24;24(3):2297. doi: 10.3390/ijms24032297 (PMC9916836; doi:10.3390/ijms24032297)
Supplement: Supplementary file 1 [file ijms-24-02297-s001.zip › Table S3.pdf]

Table S3. Oligonucleotides used in this study (5`-3`)

Gene	F/R	sequence
Primers for gene mutation		
uvrY	F	CCCTATTTCTGGAGATATTCCT GTAGGCTGGAGCTGCTTCG
uvrY	R	GCTTTTGCGTCAAACTGATCACCATATGAATATCCTCCTTAG
barA	F	CAATTTAACAGTGTGACCTTAATTGTCCCATAACGGAACTCCG TGTAGGCTGGAGCTGCTTCG
barA	R	ACAGCGTCATAAAAAGCCGATTGCTACTCGACAAGACGTCCG CATATGAATATCCTCCTTAG
ler	F	TCATTTGATTAATTGTTGGTCCTTCCTGATAAGGTCGCTAATAG CTTAAAATATTAAAGCGTGTAGGCTGGAGCTGCTTCG
ler	R	CTTTATTTTTATTATTTCATCTTCCAGCTCAGTTATCGTTATCA TTTAATTATTTCATGCATATGAATATCCTCCTTAG
Primers for identifying the gene mutants		
uvrY	F	TTCGTCGCAGGGCTTACTGTGAAA
uvrY	R	TACATCAATTTGCTGGATCTGGGC
barA	F	TGCTTCGCTGCTGTAAATCC
barA	R	TGCTATTGATACGCACTGGA
ler	F	GTGCTGGCTGTAGCTTATGTCC
ler	R	GGCTCACAATACTCATCCTCTA
Primers for pTRC99a-*uvrY*		
uvrY	F	CCGGAATTCGCCCGTATTGTTCGGGTTAATT
uvrY	R	CGCGGATCCTCACTGACTTGATAATGTCTCCGC
Primers for *uvry*-3×FLAG		
uvrY	F	CCGGAATTCTTGATCAACGTTCTACTTGTTGAT
uvrY	R	CGCGGATCCTTACTATTTATCGTCGTCATCTTTGTAGTCGATAT CATGATCTTTATAATCACCGTCATGGTCTTTGTAGTCCTGACTT GATAATGTCTCCGC
Primers for pET28a-*uvrY*		
uvrY	F	GGAATTCCATATGTTGATCAACGTTCTACTTGTTGAT
uvrY	R	CCGCTCGAGTCACTGACTTGATAATGTCTCCGC
Primers for plasmid identifying		
pKD46	F	TGAAATGCCCGTTTACCT

pKD46	R	GCCCGACTGATACGTTGA
pTrc99a	F	TGGCTGTGCAGGTCGTAAATC
pTrc99a	R	CCACACTACCATCGGCGCTA
pET28a	F	TAATACGACTCACTATAGGG
pET28a	R	GCTAGTTATTGCTCAGCGG
Primers for WT identifying		
wzx	F	CTTGGTGCTGCTCTGACATT
wzx	R	AATCGTAAGAGGGACCGTAGA
fliC	F	TCACAGTTGGCGGCGTAG
fliC	R	CATCAGCATGAAGGGTGGC
Primers for CHIP-qPCR		
P_{LEE1}	F	GCTTGGTTTTTATTCTGTTTTATTTGT
P_{LEE1}	R	ACATCTATTTCATCAAACAACCACC
$P_{LEE2/3}$	F	ATTTCCCAATAATCTTAAAAACTCTTC
$P_{LEE2/3}$	R	AATCACTCCTGTCTTCTCATCCAC
P_{LEE4}	F	CCGATTACGCATCGCACC
P_{LEE4}	R	CATTAGCCATTGGAAACTCACG
P_{LEE5}	F	ATGTTCAGCCGTTTATCGACTAC
P_{LEE5}	R	GGGATTATGACCAAGATTACCAA
P_{csrC}	F	ATGTTCAGCCGTTTATCGACTAC
P_{csrC}	R	GGGATTATGACCAAGATTACCAA
rpoS	F	GTTATCGCAGGGAGCCACA
rpoS	R	TTTTACCACCAGACGCAAGTTA
Primers for EMSA		
P_{LEE1}	F	CGCTTACCCCAATCACTTAC
P_{LEE1}	R	TCATAATAAATAATCTCCGC
$P_{LEE2/3}$	F	CATTACTGCACCAGAAGGAC
$P_{LEE2/3}$	R	AGATTCATCTGCAGGCTCTG
P_{LEE4}	F	CGCATCGCACCATTGAGAAG
P_{LEE4}	R	CATTAGCCATTGGAAACTCACG
P_{LEE5}	F	TAGTTTGCTTAATTGGTTTTCTTTGGC
P_{LEE5}	R	ACCAATAGGCATAAATATCTC
rpoS	F	GTTATCGCAGGGAGCCACA
rpoS	R	TTTTACCACCAGACGCAAGTTA

P_{csrC}	F	AAGCGGTCGTCTCCGTCAGTCAAA
P_{csrC}	R	GGGTGCTACTTTACGCCTTTGCTT
Primers for qRT-PCR		
rrsH	F	GAAAGCGTGGGGAGCAAAC
rrsH	R	ACATGCTCCACCGCTTGTG
eae	F	GACGGTAGTTCACTGGACTTCTT
eae	R	TCGCCACCAATACCTAAACG
tir	F	AAAGCAGCAGGCGAAGAGG
tir	R	TCGGCACCTGCGAATCAT
ler	F	CAGGAAGCAAAGCGACTG
ler	R	ACCAGGTCTGCCCTTCTT
escC	F	GCAATAGATGCGGCTGGAC
escC	R	TCGGCTTGTAATGGTAATATCTCG
escN	F	AGGTTTTCTTGTTGCCTTTTGA
escN	R	TCTCCATTGGTCTGCCTATGC
espB	F	AAAACTCCTCGGCAAGATGG
espB	R	AATAATCCCGCCAACCAAAG
escT	F	GCAATAGATGCGGCTGGAC
escT	R	TCGGCTTGTAATGGTAATATCTCG
Primers for verification of the RNA-seq results		
ytfl	F	GTCGGGCAACTACGAAACCATC
ytfl	R	GCTTTCTTCTTTATCTTTAGCGTCA
bssS	F	CAGACTCATCCGCTCGTAGGGT
bssS	R	TTTGGCGATTCCTGCTTCTAAT
nirC	F	TACGAGCACTCCATCGCTAACAT
nirC	R	CTTTCGGCGTAGCATACCAATAAC
adiY	F	ATTATTATTCACGGTGCTATCC
adiY	R	TTTCTTTAACAGACTTGGGCTA
ybaA	F	AGCGATGTACCGGATGGCAAAG
ybaA	R	GGCATGGACTCGCCGAACTCTT
bioC	F	ACGGCACTCCGCGAGCTGTATC
bioC	R	TGGCACTGAGCGCATCATCAAA
malF	F	GTCTGGACCGTGGTGTTCTCGC
malF	R	AAGCTCTGGTTAAACAACCCTTTG

purD	F	GATCGACAAACAGGGCAATCCG
purD	R	ATCCACCCGCAGCCATCACCAC
cyoB	F	CACCGCTATCGGGAATAGAGTAC
cyoB	R	GCACAGTGATGCCCAGGTAAAT
sdhB	F	AGGATTACACCCTGGAAGCGGAAGA
sdhB	R	GTAAACCTGGCAGCGGGCGAAT
